# Supplementary material for: OpenMM 7: Rapid development of high performance algorithms for molecular dynamics
Source: PLoS Comput Biol. 2017 Jul 26;13(7):e1005659. doi: 10.1371/journal.pcbi.1005659 (PMC5549999; doi:10.1371/journal.pcbi.1005659)
Supplement: S1 Source Code — (ZIP) [file pcbi.1005659.s002.zip › OpenMM-7.0.1-Source/docs/api-c++/generated/OpenMM.CustomExternalForce.html]

CustomExternalForce — OpenMM @OPENMM\_MAJOR\_VERSION@.@OPENMM\_MINOR\_VERSION@ documentation


# CustomExternalForce¶

*class* `OpenMM::``CustomExternalForce`¶
:   This class implements an “external” force on particles. The force may be applied to any subset of the particles in the `System`. The force on each particle is specified by an arbitrary algebraic expression, which may depend on the current position of the particle as well as on arbitrary global and per-particle parameters.

    To use this class, create a `CustomExternalForce` object, passing an algebraic expression to the constructor that defines the potential energy of each affected particle. The expression may depend on the particle’s x, y, and z coordinates, as well as on any parameters you choose. Then call `addPerParticleParameter()` to define per-particle parameters, and `addGlobalParameter()` to define global parameters. The values of per-particle parameters are specified as part of the system definition, while values of global parameters may be modified during a simulation by calling `Context::setParameter()`. Finally, call `addParticle()` once for each particle that should be affected by the force. After a particle has been added, you can modify its parameters by calling `setParticleParameters()`. This will have no effect on Contexts that already exist unless you call `updateParametersInContext()`.

    As an example, the following code creates a `CustomExternalForce` that attracts each particle to a target position (x0, y0, z0) via a harmonic potential:

    ```
    CustomExternalForce* force = new CustomExternalForce("k*((x-x0)^2+(y-y0)^2+(z-z0)^2)");
    ```

    This force depends on four parameters: the spring constant k and equilibrium coordinates x0, y0, and z0. The following code defines these parameters:

    ```
    force->addGlobalParameter("k", 100.0);
    force->addPerParticleParameter("x0");
    force->addPerParticleParameter("y0");
    force->addPerParticleParameter("z0");
    ```

    Special care is needed in systems that use periodic boundary conditions. In that case, each particle really represents an infinite set of particles repeating through space. The variables x, y, and z contain the coordinates of one of those periodic copies, but there is no guarantee about which. It might even change from one time step to the next. You can handle this situation by using the function periodicdistance(x1, y1, z1, x2, y2, z2), which returns the minimum distance between periodic copies of the points (x1, y1, z1) and (x2, y2, z2). For example, the force given above would be rewritten as

    ```
    CustomExternalForce* force = new CustomExternalForce("k*periodicdistance(x, y, z, x0, y0, z0)^2");
    ```

    Expressions may involve the operators + (add), - (subtract), \* (multiply), / (divide), and ^ (power), and the following functions: sqrt, exp, log, sin, cos, sec, csc, tan, cot, asin, acos, atan, sinh, cosh, tanh, erf, erfc, min, max, abs, floor, ceil, step, delta, select. All trigonometric functions are defined in radians, and log is the natural logarithm. step(x) = 0 if x is less than 0, 1 otherwise. delta(x) = 1 if x is 0, 0 otherwise. select(x,y,z) = z if x = 0, y otherwise.

    Methods

    |  |  |
    | --- | --- |
    | `CustomExternalForce` | Create a `CustomExternalForce`. |
    | `getNumParticles` | Get the number of particles for which force field parameters have been defined. |
    | `getNumPerParticleParameters` | Get the number of per-particle parameters that the force depends on |
    | `getNumGlobalParameters` | Get the number of global parameters that the force depends on. |
    | `getEnergyFunction` | Get the algebraic expression that gives the potential energy of each particle |
    | `setEnergyFunction` | Set the algebraic expression that gives the potential energy of each particle |
    | `addPerParticleParameter` | Add a new per-particle parameter that the force may depend on. |
    | `getPerParticleParameterName` | Get the name of a per-particle parameter. |
    | `setPerParticleParameterName` | Set the name of a per-particle parameter. |
    | `addGlobalParameter` | Add a new global parameter that the force may depend on. |
    | `getGlobalParameterName` | Get the name of a global parameter. |
    | `setGlobalParameterName` | Set the name of a global parameter. |
    | `getGlobalParameterDefaultValue` | Get the default value of a global parameter. |
    | `setGlobalParameterDefaultValue` | Set the default value of a global parameter. |
    | `addParticle` | Add a particle term to the force field. |
    | `getParticleParameters` | Get the force field parameters for a force field term. |
    | `setParticleParameters` | Set the force field parameters for a force field term. |
    | `updateParametersInContext` | Update the per-particle parameters in a `Context` to match those stored in this `Force` object. |
    | `usesPeriodicBoundaryConditions` | Returns whether or not this force makes use of periodic boundary conditions. |

    `CustomExternalForce`(*const* std::string &*energy*)¶
    :   Create a `CustomExternalForce`.

        |  |  |
        | --- | --- |
        | Parameters: |  |

        - **energy** – an algebraic expression giving the potential energy of each particle as a function of its x, y, and z coordinates

    int `getNumParticles`() *const*¶
    :   Get the number of particles for which force field parameters have been defined.

    int `getNumPerParticleParameters`() *const*¶
    :   Get the number of per-particle parameters that the force depends on

    int `getNumGlobalParameters`() *const*¶
    :   Get the number of global parameters that the force depends on.

    *const* std::string &`getEnergyFunction`() *const*¶
    :   Get the algebraic expression that gives the potential energy of each particle

    void `setEnergyFunction`(*const* std::string &*energy*)¶
    :   Set the algebraic expression that gives the potential energy of each particle

    int `addPerParticleParameter`(*const* std::string &*name*)¶
    :   Add a new per-particle parameter that the force may depend on.

        |  |  |
        | --- | --- |
        | Parameters: |  |

        - **name** – the name of the parameter

        |  |  |
        | --- | --- |
        | Returns: | the index of the parameter that was added |

    *const* std::string &`getPerParticleParameterName`(int *index*) *const*¶
    :   Get the name of a per-particle parameter.

        |  |  |
        | --- | --- |
        | Parameters: |  |

        - **index** – the index of the parameter for which to get the name

        |  |  |
        | --- | --- |
        | Returns: | the parameter name |

    void `setPerParticleParameterName`(int *index*, *const* std::string &*name*)¶
    :   Set the name of a per-particle parameter.

        |  |  |
        | --- | --- |
        | Parameters: |  |

        - **index** – the index of the parameter for which to set the name
        - **name** – the name of the parameter

    int `addGlobalParameter`(*const* std::string &*name*, double *defaultValue*)¶
    :   Add a new global parameter that the force may depend on.

        |  |  |
        | --- | --- |
        | Parameters: |  |

        - **name** – the name of the parameter
        - **defaultValue** – the default value of the parameter

        |  |  |
        | --- | --- |
        | Returns: | the index of the parameter that was added |

    *const* std::string &`getGlobalParameterName`(int *index*) *const*¶
    :   Get the name of a global parameter.

        |  |  |
        | --- | --- |
        | Parameters: |  |

        - **index** – the index of the parameter for which to get the name

        |  |  |
        | --- | --- |
        | Returns: | the parameter name |

    void `setGlobalParameterName`(int *index*, *const* std::string &*name*)¶
    :   Set the name of a global parameter.

        |  |  |
        | --- | --- |
        | Parameters: |  |

        - **index** – the index of the parameter for which to set the name
        - **name** – the name of the parameter

    double `getGlobalParameterDefaultValue`(int *index*) *const*¶
    :   Get the default value of a global parameter.

        |  |  |
        | --- | --- |
        | Parameters: |  |

        - **index** – the index of the parameter for which to get the default value

        |  |  |
        | --- | --- |
        | Returns: | the parameter default value |

    void `setGlobalParameterDefaultValue`(int *index*, double *defaultValue*)¶
    :   Set the default value of a global parameter.

        |  |  |
        | --- | --- |
        | Parameters: |  |

        - **index** – the index of the parameter for which to set the default value
        - **defaultValue** – the default value of the parameter

    int `addParticle`(int *particle*, *const* std::vector<double> &*parameters* = std::vector< double >())¶
    :   Add a particle term to the force field.

        |  |  |
        | --- | --- |
        | Parameters: |  |

        - **particle** – the index of the particle this term is applied to
        - **parameters** – the list of parameters for the new force term

        |  |  |
        | --- | --- |
        | Returns: | the index of the particle term that was added |

    void `getParticleParameters`(int *index*, int &*particle*, std::vector<double> &*parameters*) *const*¶
    :   Get the force field parameters for a force field term.

        |  |  |
        | --- | --- |
        | Parameters: |  |

        - **index** – the index of the particle term for which to get parameters
        - **particle** – [out] the index of the particle this term is applied to
        - **parameters** – [out] the list of parameters for the force field term

    void `setParticleParameters`(int *index*, int *particle*, *const* std::vector<double> &*parameters* = std::vector< double >())¶
    :   Set the force field parameters for a force field term.

        |  |  |
        | --- | --- |
        | Parameters: |  |

        - **index** – the index of the particle term for which to set parameters
        - **particle** – the index of the particle this term is applied to
        - **parameters** – the list of parameters for the force field term

    void `updateParametersInContext`(Context &*context*)¶
    :   Update the per-particle parameters in a `Context` to match those stored in this `Force` object. This method provides an efficient method to update certain parameters in an existing `Context` without needing to reinitialize it. Simply call `setParticleParameters()` to modify this object’s parameters, then call `updateParametersInContext()` to copy them over to the `Context`.

        This method has several limitations. The only information it updates is the values of per-particle parameters. All other aspects of the `Force` (such as the energy function) are unaffected and can only be changed by reinitializing the `Context`. Also, this method cannot be used to add new particles, only to change the parameters of existing ones.

    bool `usesPeriodicBoundaryConditions`() *const*¶
    :   Returns whether or not this force makes use of periodic boundary conditions.

        |  |  |
        | --- | --- |
        | Returns: | false |

High performance molecular simulation on GPUs


### Navigation

- Core Objects
- Forces
- Integrators
- Extras

©2015, Stanford University and the Authors.
|
Powered by Sphinx 1.3.1
& Alabaster 0.7.7
|
Page source
